# Arterial-jugular bulb differences in pCO_2_, lactate, serum sodium and C-reactive protein in neurocritical patients

**DOI:** 10.1186/cc13650

**Published:** 2014-03-17

**Authors:** M Canitrot, S Ugarte

**Affiliations:** 1 INDISA Clinic, Santiago, Chile

## Introduction

Several reports indicate the potential usefulness of monitoring brain metabolic parameters and their correlation with the system [1-4]. We want to establish differences and correlation in pCO_2_, lactate, serum sodium and C-reactive protein (CRP) between arterial and jugular venous bulb blood.

## Methods

An observational study. Between 1 January and 31 October 2013 we included neurocritical patients (NCP) with multimodal neuromonitoring (MMN). Daily samples of arterial blood and venous jugular bulb blood were obtained for measuring pCO_2_, lactate, serum sodium and CRP.

## Results

**Figure 1:**
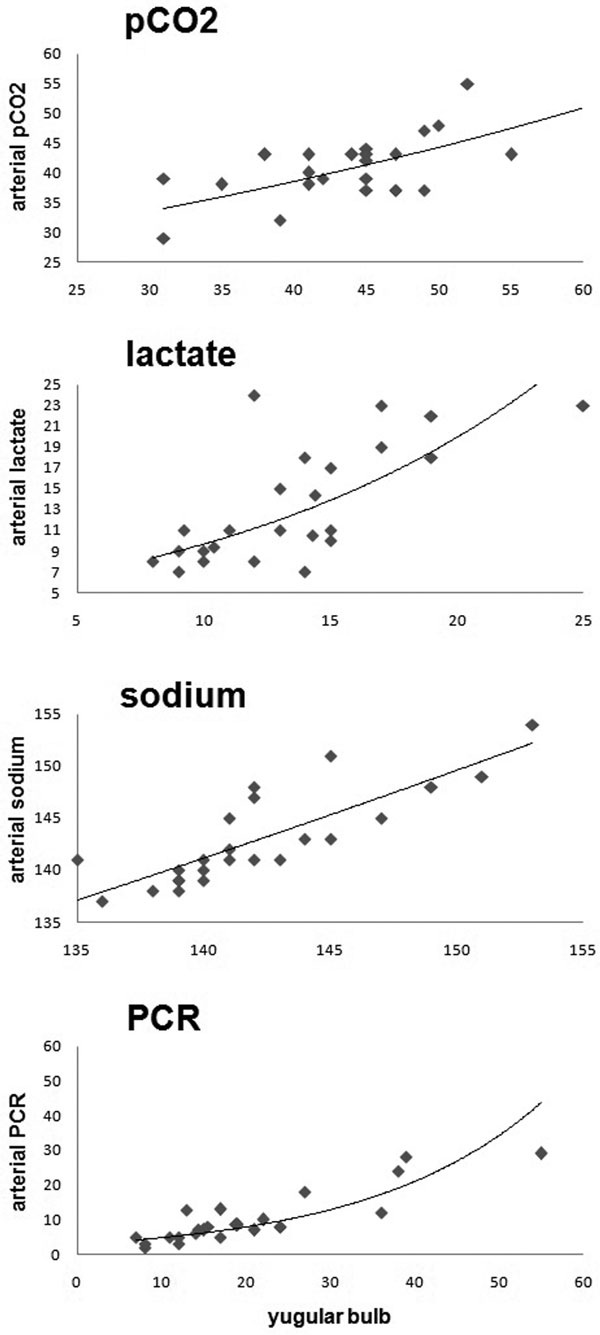


There were 45 NCP, six (13%) with MMN (five men). Mean age was 37 ± 11 years (35 to 61). Diagnostics: two TBI, two SAH, one stroke, one lupus encephalitis. APACHE II was 27 ± 6.5 (25 to 39). Glasgow Coma Scale at admission was 14 ± 4 (4 to 14). pCO_2 _(mmHg): arterial 41 ± 6.3 versus jugular 45 ± 7.4 (*r *= 0.7, *P *= 0.007). Lactate (mg/dl): arterial 11 ± 5.6 versus jugular 13.5 ± 3.9 (*r *= 0.7, *P *= 0.9). Sodium (mEq/dl): arterial 141 ± 4.5 versus 141 ± 4.4 (*r *= 0.8, *P *= 0.15). CRP (mg/dl): arterial 8 ± 7.4 versus 17 ± 11.6 (*r *= 0.9, *P *< 0.001). The correlation and trend curves are shown in Figure 1.

## Conclusion

A suitable correlation is observed for the arterial-jugular bulb in different variables. There is a significant difference in CRP and pCO_2 _values being persistently higher in the jugular, particularly for CRP. Studies are required to define its interpretation and potential usefulness.
